# Endoscopic ultrasonography-guided hepaticogastrostomy with a novel 0.018-inch guidewire and a slim-delivery metallic stent

**DOI:** 10.1055/a-2608-0713

**Published:** 2025-06-18

**Authors:** Shotaro Tsunoda, Haruo Miwa, Hiromi Tsuchiya, Kazuki Endo, Ritsuko Oishi, Yuichi Suzuki, Shin Maeda

**Affiliations:** 1 26437 Gastroenterological Center, Yokohama City University Medical Center, Yokohama, Japan; 2 Department of Gastroenterology, Yokohama City University Graduate School of Medicine, Yokohama, Japan

The utility of 22-gauge needle for endoscopic ultrasonography-guided hepaticogastrostomy (EUS-HGS) has been reported; however, a dedicated guidewire has not been developed 1 2 3 4 5 . Recently, a novel 0.018-inch guidewire (J-wire NM; J-MIT) without markers was developed to prevent guidewire stacking and a stiff shaft for smooth stent delivery. Additionally, a 7-Fr slim-delivery metallic stent (Niti-S EUS-BD system End Bare Single Flare; Taewoong Medical Co., Ltd.) has an ultra-tapered tip designed to minimize the gap with a 0.018-inch guidewire ( Fig. 1 ). Herein, we report a case in which EUS-HGS was successfully performed using a novel 0.018-inch guidewire and slim-delivery metallic stent ( Video 1 ).

**Fig. 1:**
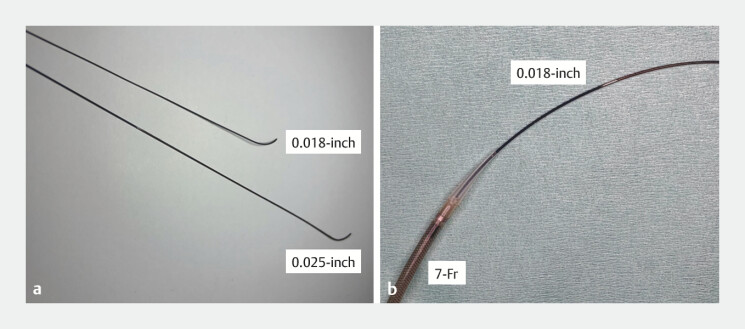
**a** A novel 0.018-inch guidewire (J-wire NM; J-MIT) without markers was developed for endoscopic ultrasound-guided hepaticogastrostomy to prevent guidewire stacking at the needle tip. **b** Novel self-expandable metallic stent with a 7-Fr slim-delivery system (Niti-S EUS-BD system End Bare Single Flare; Taewoong Medical Co., Ltd.) features an ultra-tapered tip that minimizes the gap with a 0.018-inch guidewire.

A novel 0.018-inch guidewire without markers and a 7-Fr slim-delivery metallic stent were useful for endoscopic ultrasonography-guided hepaticogastrostomy.Video 1

A 64-year-old man who had previously undergone distal gastrectomy with Roux-en Y reconstruction for advanced gastric cancer was admitted to our hospital due to obstructive jaundice caused by a recurrent tumor ( Fig. 2 ). As balloon enteroscopy could not reach the papilla of Vater due to tumor infiltration ( Fig. 3 ), EUS-HGS was attempted. The slightly dilated B3 was punctured using a 22-gauge needle (Expect Slim-line; Boston Scientific Co.) ( Fig. 4 ). After the cholangiography, the novel 0.018-inch guidewire migrated outside of the bile duct; however, it was safely removed without guidewire stacking. After re-puncture, the guidewire was successfully inserted into the bile duct. Subsequently, tract dilation was performed using a 7-Fr bougie dilator dedicated to a 0.018-inch guidewire (ES Dilator Soft; Zeon Medical, Inc.). To minimize the risk of bile leakage, the novel metallic stent (8-mm, 12-cm) with a 7-Fr slim delivery system was inserted without guidewire exchange. The metallic stent was successfully placed from the bile duct to the stomach. The patient was discharged without adverse events following the improvement of jaundice.

**Fig. 2:**
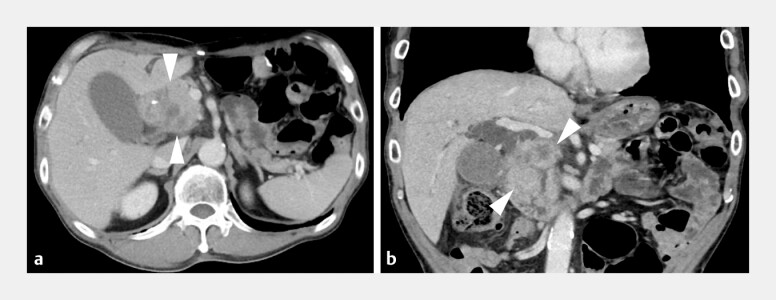
Computed tomography images show huge lymph node metastases (arrowheads) that obstruct the common bile duct and the duodenum. **a** Axis plane. **b** Coronal plane.

**Fig. 3:**
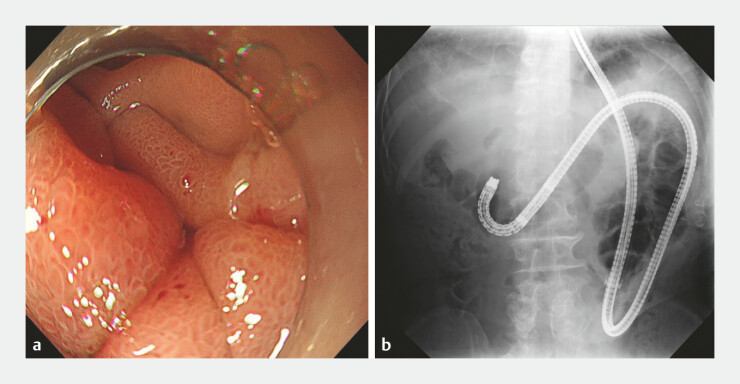
Balloon enteroscopy reveals tumor infiltration at the duodenum that prevent to reach the papilla of Vater. **a** Endoscopic image. **b** Fluoroscopic image.

**Fig. 4:**
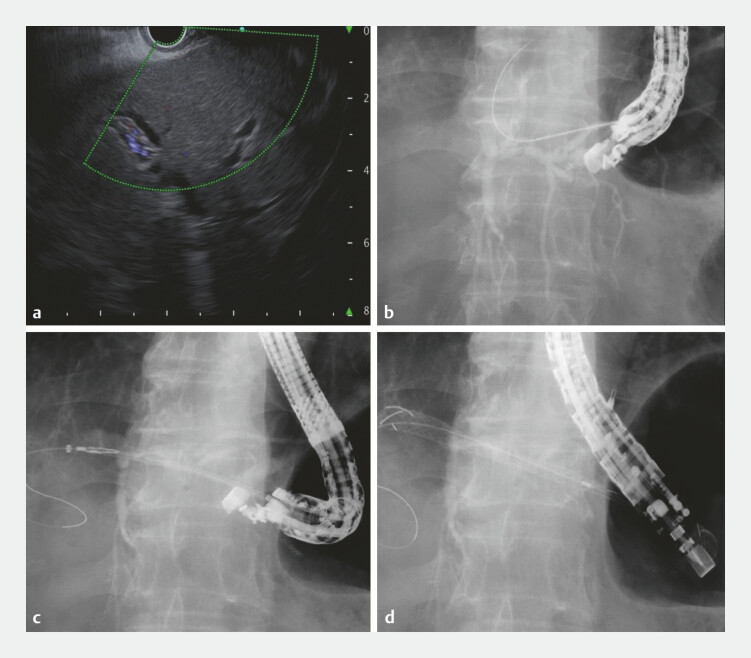
Endoscopic ultrasonography-guided hepatogastrostomy. **a** The slightly dilated bile duct is shown at segment 3. **b** Although the novel 0.018-inch guidewire migrates outside the bile duct, it can be removed without guidewire stacking. **c** After the guidewire insertion into the bile duct, a 7-Fr slim delivery of a novel metallic stent (Niti-S EUS-BD system End Bare Single Flare; Taewoong Medical Co., Ltd.) is inserted along the guidewire. **d** The metallic stent is successfully placed from the bile duct to the stomach.

To the best of our knowledge, this is the first report of EUS-HGS using a novel 0.018-inch guidewire and an ultra-tapered, slim-delivery metallic stent.

Endoscopy_UCTN_Code_TTT_1AS_2AH

## Notes

Endoscopy E-Videos https://eref.thieme.de/e-videos
*E-Videos* is an open access online section of the journal *Endoscopy* , reporting on interesting cases and new techniques in gastroenterological endoscopy. All papers include a high-quality video and are published with a Creative Commons CC-BY license. Endoscopy E-Videos qualify for HINARI discounts and waivers and eligibility is automatically checked during the submission process. We grant 100% waivers to articles whose corresponding authors are based in Group A countries and 50% waivers to those who are based in Group B countries as classified by Research4Life (see: https://www.research4life.org/access/eligibility/ ). This section has its own submission website at https://mc.manuscriptcentral.com/e-videos .
